# Nasal allergen provocation test in nasal polyposis with and without allergy

**DOI:** 10.1186/2045-7022-3-S2-O14

**Published:** 2013-07-16

**Authors:** Lien Calus, Lien Devuyst, Natalie De Ruyck, Thibaut Van Zele, Claus Bachert, Philippe Gevaert

**Affiliations:** 1 University Hospital Ghent, Upper Airway Research Laboratory, Ghent, Belgium

## Background

CRSwNP is characterised by eosinophilic inflammation and local IgE production. The amount of local tissue IgE in CRSwNP is independent of the atopic status and serum IgE of the patient. Moreover patients with CRSwNP and pollen allergy do not show prominent symptoms during season.

## Methods

Four groups of patients (n=48) underwent nasal allergen provocation test for grass pollen. We included 12 patients with allergic rhinitis based on grass allergy, 12 patients with CRSwNP without grass allergy, 12 patients with CRSwNP with grass allergy, and 12 control patients. The diagnosis of grass allergy was based on skin prick test and RAST. The test was positive based on change in nasal airflow measured by active anterior rhinomanometry and symptoms. In annex, VAS scores were performed before and after NAPT.

## Results

The nasal allergen provocation test was positive in 19 % of the patients with CRSwNP without allergy and in 54% of the patients with CRSwNP with allergy. In contrast 100% of the patients with allergic rhinitis developed a positive provocation test, whereas in the control group 8% of the patients developed a positive provocation test. CRSwNP without allergy did not show a significant increase in VAS scores of complaints. In contrast, allergic rhinitis patients and CRSwNP patients with grass allergy developed a significant increase in nasal obstruction and nasal drip. However, in allergic CRSwNP patient the symptoms after provocation were significantly lower compared to AR patients.

## Conclusion

This suggests that local IgE present in these patients are functional after allergen provocation with grass pollen. However there is a reduced reactivity after grass pollen stimulation in CRSwNP compared to allergic rhinitis. This reduced reactivity is most likely due to the polyclonality of local IgE or IgG4 blocking activity in CRSwNP.

